# Multi-objective optimisation of ultrasonically welded dissimilar joints through machine learning

**DOI:** 10.1007/s10845-022-01911-6

**Published:** 2022-02-12

**Authors:** Patrick G. Mongan, Vedant Modi, John W. McLaughlin, Eoin P. Hinchy, Ronan M. O’Higgins, Noel P. O’Dowd, Conor T. McCarthy

**Affiliations:** 1 Confirm Smart Manufacturing Research Centre, Limerick, Ireland; 2 School of Engineering, University of Limerick, Limerick, V94 T9PX Ireland; 3 Bernal Institute, University of Limerick, Limerick, V94 T9PX Ireland

**Keywords:** Machine learning, Artificial neural network, Genetic algorithm, Bayesian optimisation, Ultrasonic welding, Dissimilar materials

## Abstract

The use of composite materials is increasing in industry sectors such as renewable energy generation and storage, transport (including automotive, aerospace and agri-machinery) and construction. This is a result of the various advantages of composite materials over their monolithic counterparts, such as high strength-to-weight ratio, corrosion resistance, and superior fatigue performance. However, there is a lack of detailed knowledge in relation to fusion joining techniques for composite materials. In this work, ultrasonic welding is carried out on a carbon fibre/PEKK composite material bonded to carbon fibre/epoxy composite to investigate the influence of weld process parameters on the joint’s lap shear strength (LSS), the process repeatability, and the process induced defects. A 3^3^ parametric study is carried out and a robust machine learning model is developed using a hybrid genetic algorithm–artificial neural network (GA–ANN) trained on the experimental data. Bayesian optimisation is employed to determine the most suitable GA–ANN hyperparameters and the resulting GA–ANN surrogate model is exploited to optimise the welding process, where the process performance metrics are LSS, repeatability and joint visual quality. The prediction for the optimal LSS was subsequently validated through a further set of experiments, which resulted in a prediction error of just 3%.

## Introduction

Manufacturing of lightweight components is at the research forefront in the transportation industry (Kim et al., 2019), as a result of the ever-increasing demand for weight reduction due to increasing environmental restrictions regarding harmful emissions. Carbon fibre reinforced polymer (CFRP) composites offer superior strength to weight ratio in comparison to their traditional monolithic counterparts, making them ideal candidates for such applications. Currently more than 75% of CFRPs are manufactured using a thermoset matrix (Long, 2005), however there is increasing interest in thermoplastic composites due to their short process times, long shelf life, lack of solvent emissions during processing, and inherent recyclability (Ramaswamy et al., 2020). Therefore, the joining of thermoset matrix composites components to advanced thermoplastic matrix components is an active area of research that requires innovative joining technologies.

One such technology, showing promising potential in this space is ultrasonic welding (USW). This joining method works by converting high frequency electrical energy (typically 10–70 kHz) into high frequency low amplitude mechanical vibrations (10–250 µm). The local application of the mechanical vibrations is directed at a joint interface where a relative motion between adherends generates frictional heat to achieve a temperature sufficient to melt the adherends (Petrie, 2015). The adherends are then allowed to cool and consolidate under a controlled pressure, resulting in a fusion bond. USW is extensively used in industry due to its fast process times, low energy consumption, and is environmentally friendly (Wang et al., 2017). However, degradation of the matrix and fibre re-orientation, resulting in low-performing components are drawbacks when joining fibre composite materials. It has been shown that joint quality shows a strong dependence on weld input parameters and the relationships are extremely non-linear (Mongan et al., 2021). Therefore, it is important to identify the appropriate weld input parameters during the process development stage. Moreover, the complex non-linear relationships are difficult to model using traditional techniques.

Recent advances in the digitisation of the manufacturing industry have led to an increasing interest in the use of machine learning in intelligent manufacturing technologies, including welding systems (Wang et al., 2020). Machine learning uses computational algorithms to convert empirical data into predictive models (Edgar & Manz, 2017). Artificial neural networks (ANN), a class of machine learning algorithms, are a data-driven modelling method with universal approximation capabilities and flexible structure that enable the method to capture complex non-linear behaviours (Markopoulos et al., 2008; Shokry & Espuña, 2018). ANN’s have unprecedented utility in predicting the USW process due to their ability to model the complex non-linear relationship between process parameters and joint performance. Various researchers have investigated ANN modelling of welding processes, Liu et al. (2020) optimised a laser welding process using an ANN trained on sixteen samples from a Taguchi experimental design, concluding the process was optimised by the reduction in porosity and the increase in strength relative to the observed experimental data. Tafarroj and Kolahan (2018) compared the performance between ANN and regression models for a gas tungsten arc welding application where the training data consisted of twenty-seven experimental samples. The study found ANN modelling provided a more accurate prediction. Similarly, McDonnell et al. (2021) compared the performance of Gaussian process regression (GPR), support vector machines (SVM), and ANN’s for the multi-objective optimisation of a laser machining process identifying ANN as the superior machine learning method. Seyyedian Choobi et al. (2012) used forty-one training samples to train an ANN with fifteen, twenty and twenty-five neurons in the first, second and third hidden layers, respectively. The model’s predictions of welding deflections were validated by finite element simulations, producing a mean error of 0.66%. Within the present context of predicting the strength of USW joints, Pradeep Kumar and Divyenth (*^2020^) trained an ANN to predict the performance of copper wire joined by USW to copper sheet using twenty-seven training samples and produced a correlation coefficient of 0.96 between predicted and measured strength. However, the model was not assessed on test data. Mongan et al. (2020) developed an ANN to predict the weld quality for USW aluminium 5754, achieving a correlation coefficient of 0.98 between predicted and actual values for lap shear strength (LSS). Zhao et al*.* (2017) developed an ANN on twenty-seven training samples to predict the strength of aluminium 6061 joined to A36 steel, producing a correlation coefficient of 0.998 between predicted and measured results.

While it is clear that USW has been reported for several different alloy combinations, it has not been widely used for polymer composite materials. Thus, understanding of the influence of welding parameters on joint performance for polymer composites is limited, and effective methods are not currently available to predict joint performance (Sun et al., 2021; Wang et al., 2017). It should also be noted that none of the studies outlined above optimised the process or assessed repeatability within the process and so gaps are evident in the open literature. This study aims to deliver a multi-objective optimisation model that is capable of predicting, with a high degree of accuracy, the maximum achievable LSS of a USW carbon fibre reinforced polymer dissimilar composite joint, while ensuring a repeatable process and no process induced defects.

Despite their promising results, ANNs are commonly classified as “black box” models (Oliveira et al., 2015) since, for example in the context of joint strength prediction, they do not explain the underlying bonding mechanisms that give rise to increased joint performance. Furthermore, the model’s performance and its training convergence have a strong dependency on the selection of the model hyperparameters (Le-Hong et al., 2021). Therefore, identifying an appropriate set of hyperparameters is essential to obtain acceptable results. To this intent, in this study, Bayesian optimisation (BO) was implemented to optimise the selection of the optimal ANN hyperparameters. Various researchers have taken a similar approach, where Shin et al. (2020) optimised neural network hyperparameters using BO for predicting NO_x_ in a diesel engine and Wu et al. (2019) demonstrated that using BO for hyperparameter tuning of machine learning models increases the robustness of the models. Snoek et al. (2012) also demonstrated the benefits of using BO for hyperparameter tuning of various machine learning models concluding BO surpasses a human expert at selecting optimal hyperparameters.

The objective of this work is to demonstrate the benefits of using machine learning approaches for the multi-objective optimisation of a complex USW process for the joining of dissimilar materials. The approach is as follows: three repetitions of a 3^3^ design of experiment (DoE) parametric study are first conducted to acquire insight into the joining process. The experimental data are then analysed to identify key contributors to the joints lap shear strength (LSS), the repeatability of the process, and process induced defects. The experimental data are then used to develop a robust hybrid genetic algorithm-artificial neural network (GA–ANN) predictive model in conjunction with BO for hyperparameter tuning. The GA-ANN model is then used to predict the weld input parameters to achieve the maximum LSS for a defect free and repeatable process. The prediction is then subsequently validated through experimentation.

## Experimental procedure

### Materials

**Fig. 1:**
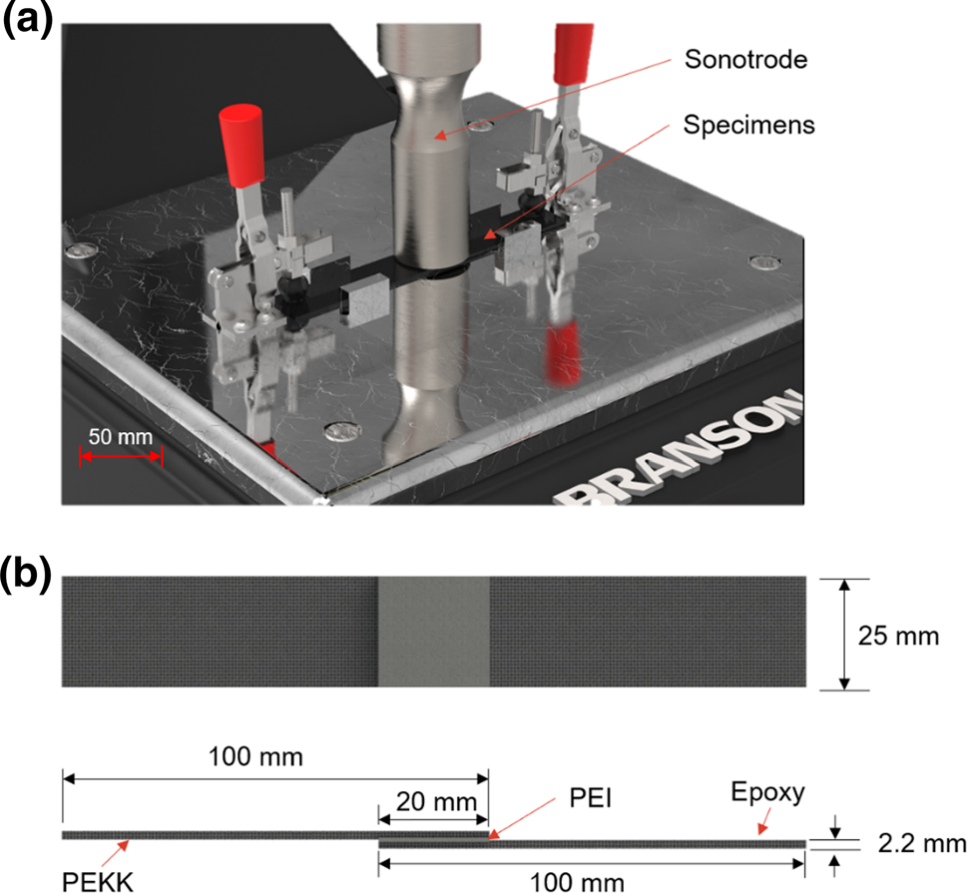
**a** Schematic of welding configuration and **b** welded specimen geometry.

The materials investigated in this study are Tenax^®^-E HTS45 carbon fibre reinforced polyetherketoneketone (CF/PEKK) bonded to Hexcel IM7 carbon fibre reinforced HexPly^®^ 8552 epoxy (CF/epoxy). PEKK is a semi-crystalline thermoplastic in the polyaryletherketone (PAEK) family, with high heat resistance, chemical resistance, and the ability to withstand high mechanical loads (ASTM International, 2012). The glass transition temperature (*T*_*g*_) of PEKK is 162 °C (Li & Strachan, 2019). Epoxy is a thermoset polymer with excellent adhesion, chemical and heat resistance, good mechanical properties, and very good electrical insulating properties (May, 1988). The *T*_*g*_ of the epoxy used in this study is 154 °C (HEXCEL Corporation, 2020). Polyetherimide (PEI) is compatible with both PEKK and epoxy and was used as an energy director at the joint interface to promote the formation of strong joints (Villegas & Moorleghem, 2018). A 0.125 mm thick Sabic Ultem 1000 PEI strip was co-cured with the CF/epoxy sheet before welding. A description of the laminates used in this study is shown in Table 1, while a schematic of the welding configuration and the welded test specimen geometry is provided in Fig. 1.

**Table 1:** Description of the laminates used during welding

Material	Supplier	Stacking sequence	No. of plies	Matrix	Thickness (mm)
CF/PEKK	Teijin GmbH	${\left[0^\circ \right]}_{16}$	16	Thermoplastic	2.2
CF/Epoxy	Hexcel	$\left[-45^\circ /0^\circ /+45^\circ /90^\circ \right]$	16	Thermoset	2.2

### Ultrasonic welding

An USW machine consists of five subsystems: (1) power supply with an integrated controller, (2) transducer, (3) a booster, (4) a sonotrode, and (5) a pneumatic cylinder. During welding, the controller monitors all subsystems through integrated sensors to control the process. The power supply provides high frequency electrical energy to an ultrasonic stack composed of a transducer, booster and sonotrode. The core of the transducer is a lead-zirconate-titanate electrostrictive element that expands and contracts when subjected to alternating voltage. Therefore, through the piezoelectric effect, the transducer converts the high frequency electrical energy into mechanical oscillations. The booster then amplifies the vibrations based on a gain ratio before the sonotrode transmits the ultrasonic oscillations through the weld substrates and oscillations are finally imposed to the joint interface. Ultrasonic welding of polymer composites can be segregated into two phases, a vibration phase in which sufficient heat is generated through surface friction and viscoelastic heating to melt the matrix allowing it to flow, and a solidification phase where the adherends are allowed to cool under a controlled pressure, achieving consolidation (Koutras et al., 2019).

In this study, the lap joints were manufactured using a Branson 2000Xdt ultrasonic welder equipped with a 20 kHz power supply and circular sonotrode with a diameter of 40 mm. The process was controlled using energy control mode which terminates the vibration portion of the weld when the joint interface absorbs the preselected welding energy. The parameters varied in the DoE are: welding energy, vibration amplitude and welding force. Their respective level combinations can be seen in Table 2. The effect of welding input parameters on joint performance metrics is material dependent, so existing process insight is not transferable to the material combination examined here. Therefore, a wide parameter space was selected to provide data to characterise the process over a large application field. The LSS of the joints was determined using a Zwick 100 kN tensile tester with a crosshead speed of 13 mm/min in accordance with ASTM D 5868 (ASTM International, 2001). To assess process repeatability and to eliminate the effect of unknown variables, three DoE repetitions were conducted and the run sequence was randomised.

**Table 2:** Parameter and level combinations for the DoE

Parameters	Level 1	Level 2	Level 3
Welding energy (kJ)	1.0	1.75	2.5
Vibration amplitude (µm)	85	100	115
Welding force (N)	400	800	1200

### Process repeatability and process induced defects

The DoE data are presented in Table 3. An average of the three experimental repeats is taken to represent the LSS. During experimentation it was identified that for certain parameter combinations the process has poor repeatability, and/or the welding process induces defects in the weld area. Therefore, additional modelling parameters were required. This study incorporated a relative standard deviation (RSD) to quantify the variation in the joints’ LSS performance. The LSS response window is large (2.59–25.98 MPa), so RSD is used due to its ability to provide a relative metric for process repeatability. RSD is outlined as follows:

1 $$RSD=100 \times SD/\overline{x }$$

where $\overline{x}\;{\text{is}}\;{\text{the}}\;{\text{average}}\;{\text{result}},\;{\text{and}}\;SD$ is the standard deviation formulated as follows:

2 $$SD= \frac{1}{\sqrt{n-1}}\sqrt{\sum_{i=1}^{n}({x}_{i}-\overline{x }{)}^{2}}$$

where *n* is the number of values (three for this analysis).

**Table 3:** Experimental results with categorised defects and repeatability

Physical run number	Welding energy (kJ)	Vibration amplitude (µm)	Welding force (N)	LSS (MPa)	RSD (%) [repeatability class]	Defect class
29/30/69	1	85	400	12.90	24.41 [2]	2
39/40/74	1	85	800	13.50	52.99 [3]	2
25/26/67	1	85	1200	22.04	13.83 [2]	1
37/38/73	1	100	400	10.79	54.87 [3]	3
35/36/72	1	100	800	17.43	21.47 [2]	1
21/22/65	1	100	1200	23.41	11.71 [2]	2
45/46/77	1	115	400	14.48	8.35 [1]	1
31/32/70	1	115	800	17.38	8.52 [1]	1
49/50/79	1	115	1200	6.04	18.90 [2]	1
21/52/80	1.75	85	400	22.26	9.98 [1]	2
15/16/62	1.75	85	800	10.56	28.80 [2]	2
19/20/64	1.75	85	1200	2.59	35.98 [3]	3
9/10/59	1.75	100	400	16.38	13.15 [2]	2
23/24/66	1.75	100	800	19.71	6.45 [1]	2
3/4/56	1.75	100	1200	25.98	1.01 [1]	3
41/42/75	1.75	115	400	17.12	25.31 [2]	1
17/18/63	1.75	115	800	16.26	24.84 [2]	2
11/12/60	1.75	115	1200	16.22	14.80 [2]	1
7/8/58	2.5	85	400	15.48	24.95 [2]	3
1/2/55	2.5	85	800	14.33	18.74 [2]	1
47/48/78	2.5	85	1200	5.06	95.03 [3]	3
43/44/76	2.5	100	400	9.70	25.50 [2]	3
33/34/71	2.5	100	800	6.62	70.70 [3]	3
53/54/81	2.5	100	1200	6.79	73.03 [3]	3
13/14/61	2.5	115	400	11.59	78.05 [3]	3
27/28/68	2.5	115	800	12.46	5.76 [1]	3
5/6/57	2.5	115	1200	7.57	82.76 [3]	2

To minimise the complexity of the optimisation problem, this study categorised process repeatability into three classes, as outlined in Table 4.

**Table 4:** Classification of process repeatability

Class	RSD (%)
1	0 < RSD ≤ 10
2	10 < RSD ≤ 30
3	30 < RSD

**Fig. 2:**
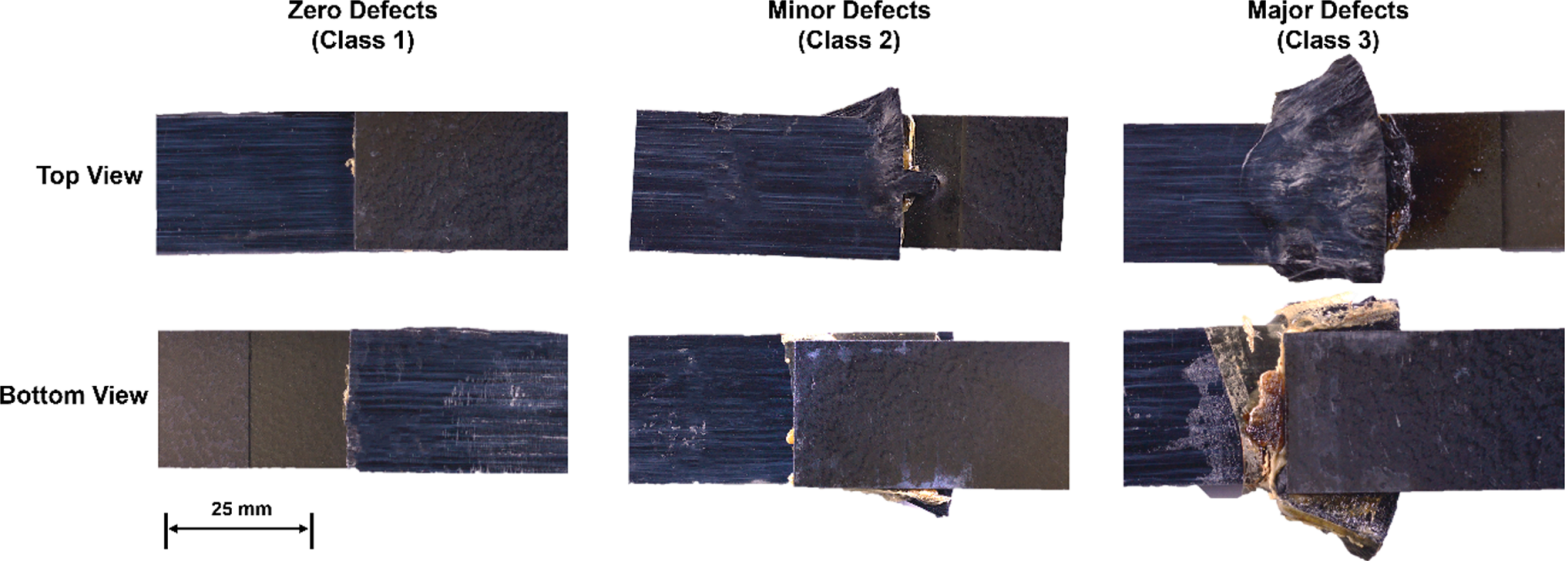
Classification of joint quality based on visual assessment

When conducting the DoE, it was noted that although some parameter combinations performed well (under LSS criteria), there were unacceptable visual defects present on the joint (matrix degradation and fibre pushout). Process-induced defects are a key concern when joining dissimilar materials and add additional complexity to the optimisation process. Therefore, to prevent mapping defective relationships, a visual quality classification method was devised to account for process induced defects, as outlined in Fig. 2, i.e., Class 1 corresponding to zero visual defects, Class 2 corresponding to minor visual defects, Class 3 corresponding to major visual defects.

### Experimental data analysis

**Fig. 3:**
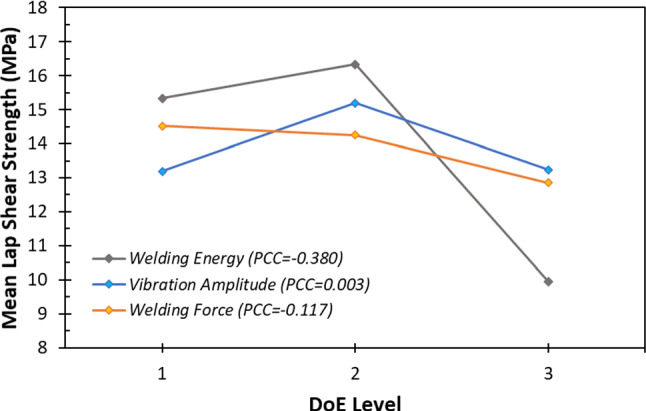
Main effects plot and Pearson’s correlation coefficient (PCC) between input parameters and LSS

Main effects plots and Pearson’s correlation coefficient (PCC) have been used to analyse the DoE data. A main effects plot illustrates the response for the different input levels (as in Table 2), while the PCC quantifies the statistical strength of a relationship between variables (Lee Rodgers & Alan Nice Wander, 1988). An effect is declared as the variation in the response as a result of changing a factor from one level to another (Jawaid et al., 2018). Figure 3 presents a main effects plot of the DoE with associated PCC. It is apparent from this figure that the process was characterised over a large application field, indicated by the regions of positive correlation between DoE Levels 1 and 2, and the negative regions of correlation between DoE Levels 2 and 3 for the welding energy and vibration amplitude. The variation in LSS caused by welding energy is larger than those caused by other parameters. However, welding energies above DoE level 2 (1.75 kJ) produce joints of reduced LSS performance, which is attributed to process induced defects (thermal degradation). A similar relationship can be seen between vibration amplitude and the LSS—when vibration amplitude exceeds 100 µm (level 2), the LSS decreases.

**Fig. 4:**
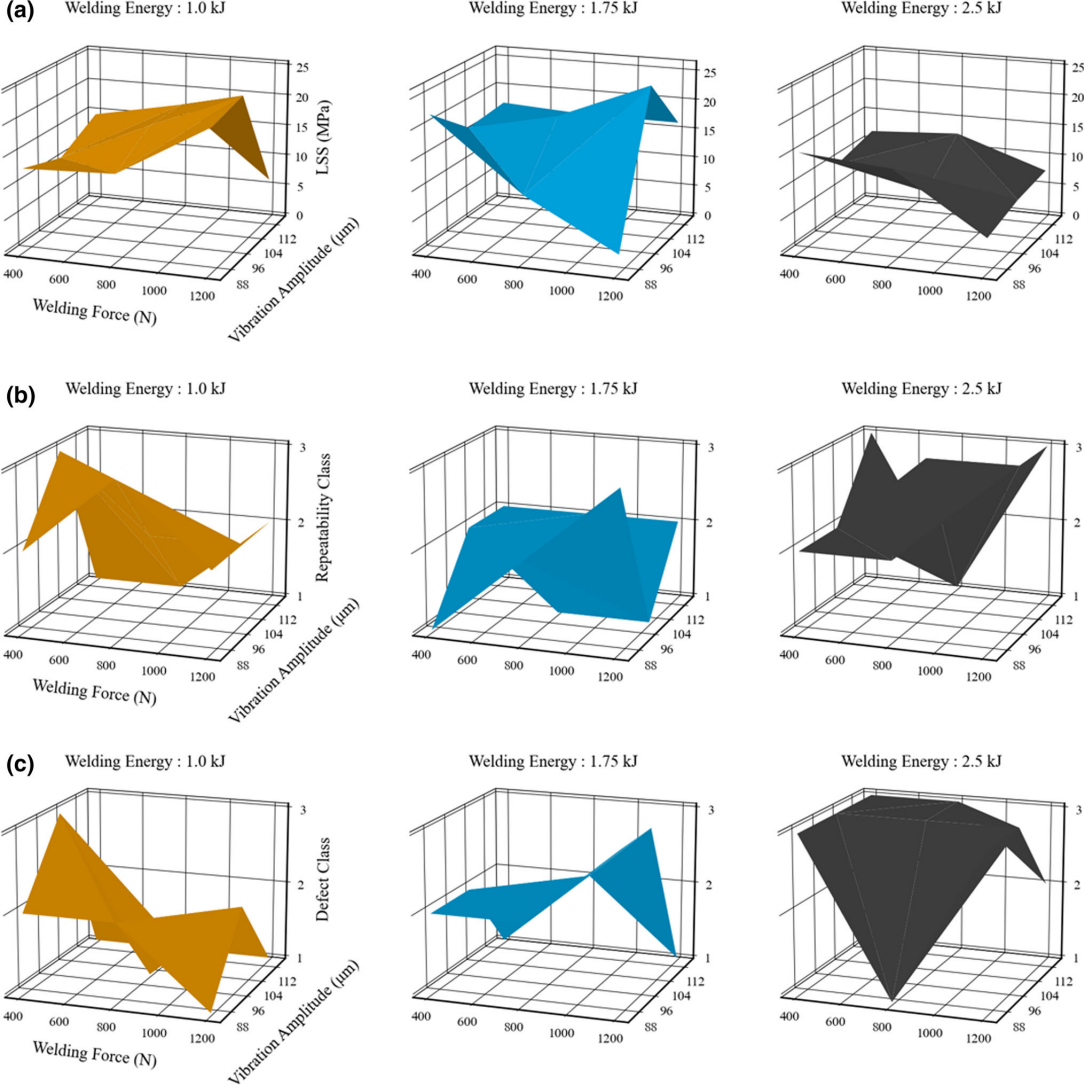
Surface plots displaying the results of the DoE, **a** distribution in LSS, **b** repeatability of the process and **c** the variation in defect class

Figure 4a illustrates the dependence of the LSS on the process input parameters. It is seen that the LSS response is non-linear with respect to input parameters, characterised by no consistent flat regions and large fluctuations in values of LSS (from 5 to 25 MPa). Figure 4a shows that at a welding energy of 1 kJ, high values of LSS are achieved at low vibration amplitudes and high welding force. However, similar trends are not observed at welding energies of 1.75 kJ, and 2.5 kJ. Figure 4b highlights process repeatability for the different input parameters. The lack of process repeatability for certain process parameters is assumed to be the sharp melting temperatures of the CF/PEKK (Wang et al., 2021). Therefore, identifying the correct process parameters is crucial to establish a stable process. Figure 4c displays the defect classes identified for the different process parameters. It is seen that increasing the welding energy increases the density of defects. It may also be noted in Fig. 4c that high values of vibration amplitude are associated with fewer defects. This is attributed to the reduction in process time at higher vibration amplitude, preventing thermal degradation of the matrix because higher amplitudes exert the required energy quicker at the joint interface. In general, for the parameter ranges chosen a significant number of defects have been detected.

As indicted in “Ultrasonic welding” section, a wide parameter range was selected to characterise the process over a large application field. However, this has shown to produces uncertainties within the dataset, thus increasing the complexity of the optimisation task. Therefore, optimising the process using traditional techniques would require, at a minimum, one additional DoE with refined parameter ranges to capture the local responses in the region that has a high potential to contain the global optimum. This would lead to high experimental waste (adherends, time, resources). As an alternative, this study optimises the multi-objective problem through machine learning.

## Machine learning

Due to the complex and non-linear nature of the process, the authors considered multiple machine learning methods such as ANNs, gaussian process regression (GPR), random forest (RF), and support vector machines (SVM). However, published studies have demonstrated the excellent capabilities of ANNs in accurately capturing the non-linearity in the USW process (Mongan et al., 2020, 2021; Pradeep Kumar & Divyenth, 2020; Zhao et al., 2017). Therefore, this study adopts an ANN modelling approach.

### Predictive modelling procedure

**Fig. 5:**
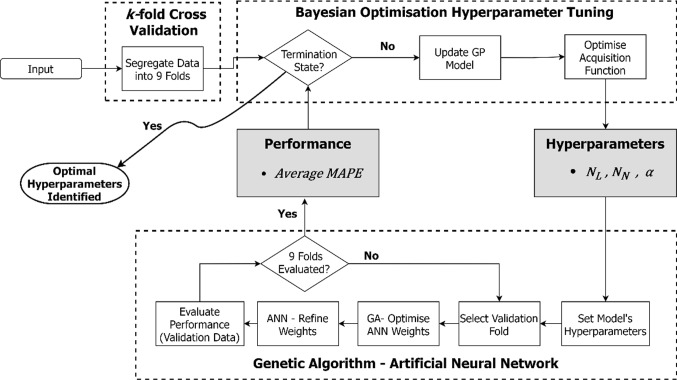
Schematic displaying process flow for generating the supervised optimisation model

The optimisation of USW process is challenging due to the number of process evaluations which are severely limited by time and the cost associated with the adherends. The relatively small dataset adds additional complexity to the process resulting in a high dependency of the prediction performance on the ANN’s hyperparameters. Furthermore, the drawbacks of ANN modelling such as local minima convergence, poor natural global search ability and the tendency to overfit on the training data, must be mitigated. For the aforementioned reasons, in this study, the ANN is combined with a genetic algorithm (GA) to initialise the networks weights, allowing for random exploration of the loss surface through gradient free optimisation. The GA–ANN model’s hyperparameters are optimised using BO, where the BO objective function is used to maximise the GA–ANN’s performance on validation data by varying the models hyperparameters. To ensure that the GA–ANN provides a true representation of the model’s ability to generalise, this study implements k-fold cross validation. The procedure for developing the model is illustrated in Fig. 5. Each aspect of the flowchart is next discussed in detail.

### Bayesian optimisation

BO is an efficient method for the global optimisation of unknown objective functions by iteratively evaluating the function at carefully chosen locations. Thus, it is ideal for hyperparameter tuning (Snelson, 2007). BO combines prior distribution of the function *f*(*x*) with sample information to obtain the posterior of the function (Wu et al., 2019). The posterior information is used to identify the location of the function maximum, according to a particular criterion. In this study the criterion is the negative average *k*-fold cross validation MAPE. The BO procedure for hyperparameter tuning is summarised by the following pseudo code:
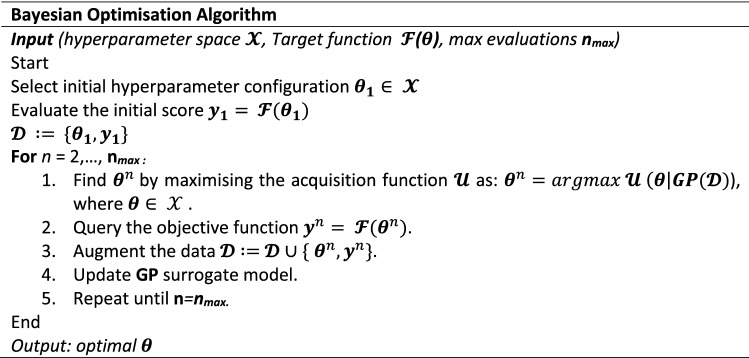


BO algorithms aim to find the global maximiser ${{\varvec{\theta}}}^{\boldsymbol{*}}$ by leveraging two main components: (i) a probabilistic function (surrogate) model, used to approximate the unknown objective function $\mathcal{F}$**,** and (ii) the acquisition function $\mathcal{U}$**,** which determines the next set of hyperparameters to query. This study assumes the optimisation function to be Gaussian distributed. Therefore, the prior distribution of the hyperparameters can be determined using GPR. The GPR model used in this study is based on Rasmussen and Williams (2006), where a detailed description can be found. The kernel function is critical to the accuracy of the GPR prior (AlBahar et al., 2021), for that reason, this study used a Matérn covariance kernel defined as follows:

3 $$Mat\acute{e} rn(x,{x}^{{\prime}})= \frac{1}{{2}^{v-1 }\Gamma \left(v\right)}{ \left(\sqrt{2v}\frac{\left|x-{x}^{{\prime}}\right|}{l}\right)}^{2} {\mathcal{B}}_{v}\left(\sqrt{2v}\frac{\left|x-{x}^{{\prime}}\right|}{l}\right),$$

where $l$ is the correlation length parameter, $\Gamma$ is the standard Gamma function, ${\mathcal{B}}_{v}$ is the modified Bessel function, (Tolba et al., 2019), and $v$ is a hyperparameter that controls the smoothness of the resulting function.

This study implements the expected improvement acquisition function because of its ability to automatically balance the trade-off between ‘exploration’ and ‘exploitation’ (Archetti & Candelieri, 2019). The hyperparameters optimised were the number of hidden layers (*N*_*L*_), the number of neurons (*N*_*N*_), and the initial learning rate (α). Table 5 provides the hyperparameter bounds ([min, max]) and their respective values for the best performing model.

**Table 5:** Hyperparameter bounds for BO

Hyperparameter	Bound	Optimal
*N*_*L*_	[1, 4]	2
*N*_*N*_	[4, 30]	20, 20
α	[0.0001, 0.5]	0.054

### *k-fold* cross validation

ANN modelling has a tendency to over-fit on training data. To mitigate this phenomenon, validation data is incorporated to control the training process while providing insight into the model’s ability to generalise (Vidyasagar, 2003). However, as a result of the relatively small dataset selecting one particular subset as validation data can lead to biased estimates of the model’s ability to generalise. Therefore, this study implements *k-fold* cross validation with an early stopping technique, which divides the dataset into several ‘folds’ of equal size (*k* = 9 for this study). Each fold is selected in turn as validation data, with the remaining folds used for training. The process is repeated until all folds have been assessed once, and then the average of all prediction accuracies is used to represent the ANN’s performance. The early stopping technique terminates training when the validation error starts to increase. To ensure training does not terminate prematurely due to a temporary fluctuation in validation error, a patience interval is incorporated to provide the model a further ten epochs to reduce the validation error, and if successful, training continued.

### Genetic algorithm (GA)

A GA is a robust optimisation algorithm inspired by the process of biological evolution (Kapoor, 2019). This study exploits the global search ability of the GA with the local search of the ANN. GAs are used to solve linear and non-linear optimisation problems by exploring regions of the parameter space through crossover, mutation, and a selection function based on a fitness value. In this study, after a random population of twelve models is generated and their performance is evaluated, the best eight performing models are ranked and progress to the crossover stage. The crossover stage pairs the models in groups of two and creates an offspring with half the weights from each model. The role of the mutation phase is to provide diversity in the population by randomly changing 10% of the weights for a randomly chosen model. When evaluating the individual model’s performance, the GA vector representation is mapped to the ANN matrices representation to emulate the ANN prediction process. Once all operations are performed, the process is repeated in a new generation that is genetically better than the previous. The process continues until the pre-set maximum number of generations (2500) is reached. The procedure for integrating the GA and ANN in this study is illustrated in Fig. 5 and the GA hyperparameters are presented in Table 6.

**Table 6:** Hyperparameters values of the GA

Hyperparameter	Value
Number of models	12
Number of mating parents	8
Mutation rate (%)	10
Generations	2500

### Artificial neural network (ANN)

An ANN consists of layers of neurons interconnected to each other by weights. The first layer is defined as the input layer, the last layer is the output layer, and the remaining layers in between are the hidden layers. Layers are composed of a number of compute neurons. Each compute neuron is characterised by its input, bias, activation function, and output. ANNs are trained in an iterative approach, whereby the prediction errors are evaluated by a metric; a training algorithm then updates the connecting weights and bias to reduce the succeeding error. The process continues until the model converges, based on a termination criterion. ANN’s have a high capacity to model complex non-linear problems such as the USW process under investigation. However, the accuracy of the solution depends on the network’s hyperparameter configuration. Key hyperparameters affecting the performance of ANNs are: the number of layers (*N*_*L*_), the number of compute neurons in each layer (N_N_), the initial learning rate (α), the activation function, the training algorithm, and the number of training epochs (Uguz & Ipek, 2020), where N_L_, N_N_ and α were optimised using BO. The activation function is a key element that enables ANNs to resolve complex nonlinear relationships. This study selected the rectified linear unit (ReLU) because of its computational simplicity in achieving a high degree of accuracy and its fast compute times in comparison to other activation functions, such as Tanh and Sigmoid (Krizhevsky et al., 2012). There is a large unit difference within the input data, which can lead to increased computational complexity. Therefore, the data was normalised into the values between [0, 1]. The evaluation metric quantifies the ANN’s performance and is a key tool in comparing the performance of different ANNs. The problem being investigated in this current work is multi-objective with a large unit difference within the output data. Therefore, to ensure the model’s accuracy is consistent across all outputs the mean absolute percentage error (MAPE) is selected as the evaluation metric, due to its ability to provide a relative error. The MAPE metric is defined as:

4 $${\text{MAPE}}= \frac{1}{n}\sum_{i=1}^{n}\left(\frac{{\text{actual}}\,{\text{value}} - {\text{predicted}}\,{\text{value}}}{{\text{actual}}\,{\text{ value}}} \times 100\right).$$

The model’s weights and biases were refined using the adaptive moment (Adam) estimation algorithm. Adam is an algorithm for first-order gradient-based optimisation that is computationally efficient while having small memory requirement (Kingma et al., 2015). Using the Adam algorithm, each parameter being optimised has its own adaptive learning rate which is determined by calculating the first-moment and second-moment estimations of the gradient. The Adam algorithm and the associated parameters used in this study are summarised by the following pseudo code:
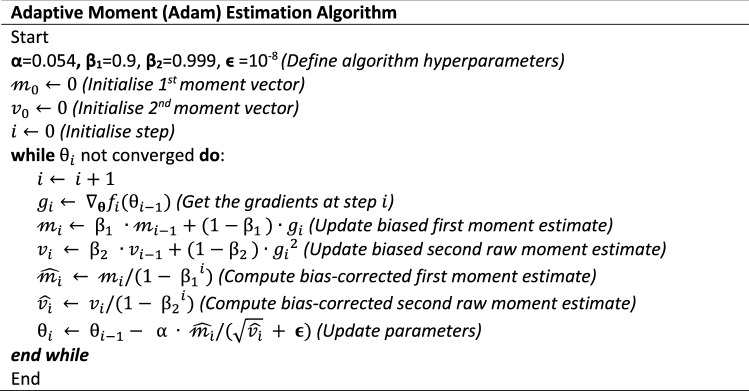


### Final model and process parameter optimisation

**Fig. 6:**
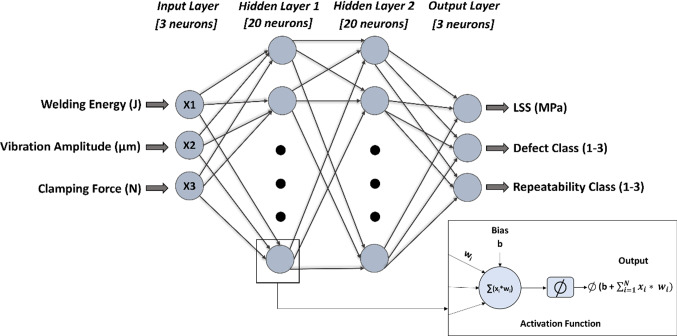
The optimised ANN configuration and the ANN process of prediction through a single compute neuron

Figure 6 illustrates the ANN configuration for the best performing model (*N*_*L*_ = 2; *N*_*N*_ = 20, 20). As indicated, the metric to identify the optimal hyperparameters was the average *k*-fold cross validation error. Therefore, to ensure performance consistency when predicting the parameter space, each trained instance of the model was stored (i.e., nine instances as *k* = 9) and then combined to create an ensemble prediction approach. The ensemble prediction represents the mean of all nine base learners. Ensemble learning combines multiple diverse base learners to obtain robustness and superior generalisation accuracy compared with any of the constituent learners (Shaikhina & Khovanova, 2017). In the USW process the input parameters take integer values, therefore, the parameter space is discrete. Once trained, interrogating the GA–ANN ensemble to retrieve LSS, defect and repeatability predictions for the entire bounded parameter space took less than 180 s. A similar approach was implemented by McDonnell et al. (2021) when optimising a multi-objective laser machining process, where the optimised process was superior to the observed DoE data. In this study, the predictions were formatted to return the input parameters corresponding to the maximum LSS achievable when the model predicts the process to produce zero defects while having class one repeatability.

## Results: multi-objective optimisation model

The average *k-fold* validation MAPE for the optimised configuration is 3.74, which is an assessment of the model’s ability to generalise. The low MAPE value indicates the model’s hyperparameters were accurately optimised and the model can predict with a high degree of accuracy unseen parameter combinations. The resulting ensemble model demonstrated 100% accuracy in predicting the correct defect and repeatability classes for both training and validation data.

**Fig. 7:**
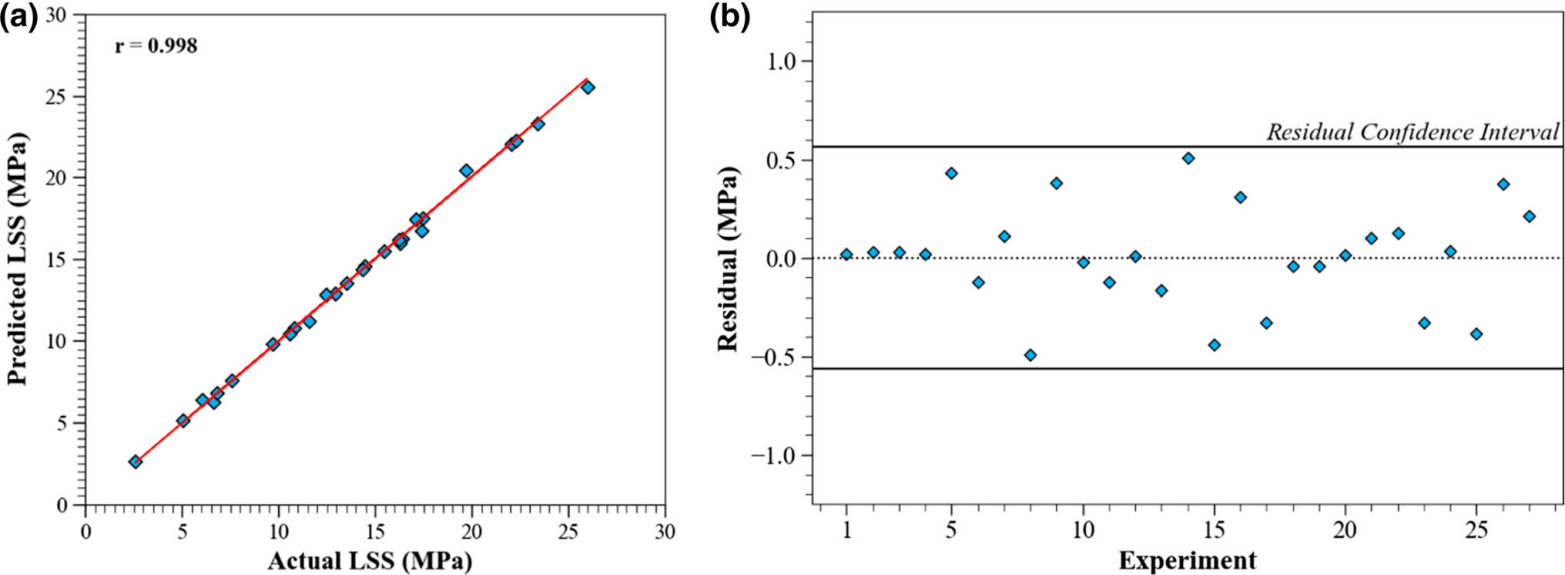
Summary of predictions. **a** Regression analysis, **b** residual analysis

To evaluate the LSS prediction accuracy, a regression and residual analysis was performed and is displayed in Fig. 7. The results of the regression analysis shown in Fig. 7a indicate that there is a very high PCC (= 0.998) between the experimental LSS values and the corresponding predicted values, showing excellent agreement. The residual analysis shown in Fig. 7b highlights the magnitude of the prediction errors and the residual confidence region. The residual is defined as follows:

5 $${r}_{i}={\text{experimental}}_{i}-{\text{predicted}}_{i}.$$

The 95% confidence interval is calculated as follows:

6 $$\overline{r } \pm 1.96 \times SD.$$

where $\overline{r}\;{\text{is}}\;{\text{the}}\;{\text{residual}}\;{\text{mean}}\;{\text{and}}\;SD$ is the standard deviation calculated using Eq. (2). The residual mean, SD and confidence interval are 0.0085 MPa, 0.29 MPa, and ± 0.56 MPa, respectively. In comparison to the joint’s strength the magnitude of the SD is small, and the residual mean is close to zero, indicating the model has a high degree of accuracy. It is evident from Fig. 7b that all prediction residuals are within the residual confidence region of 95%.

**Fig. 8:**
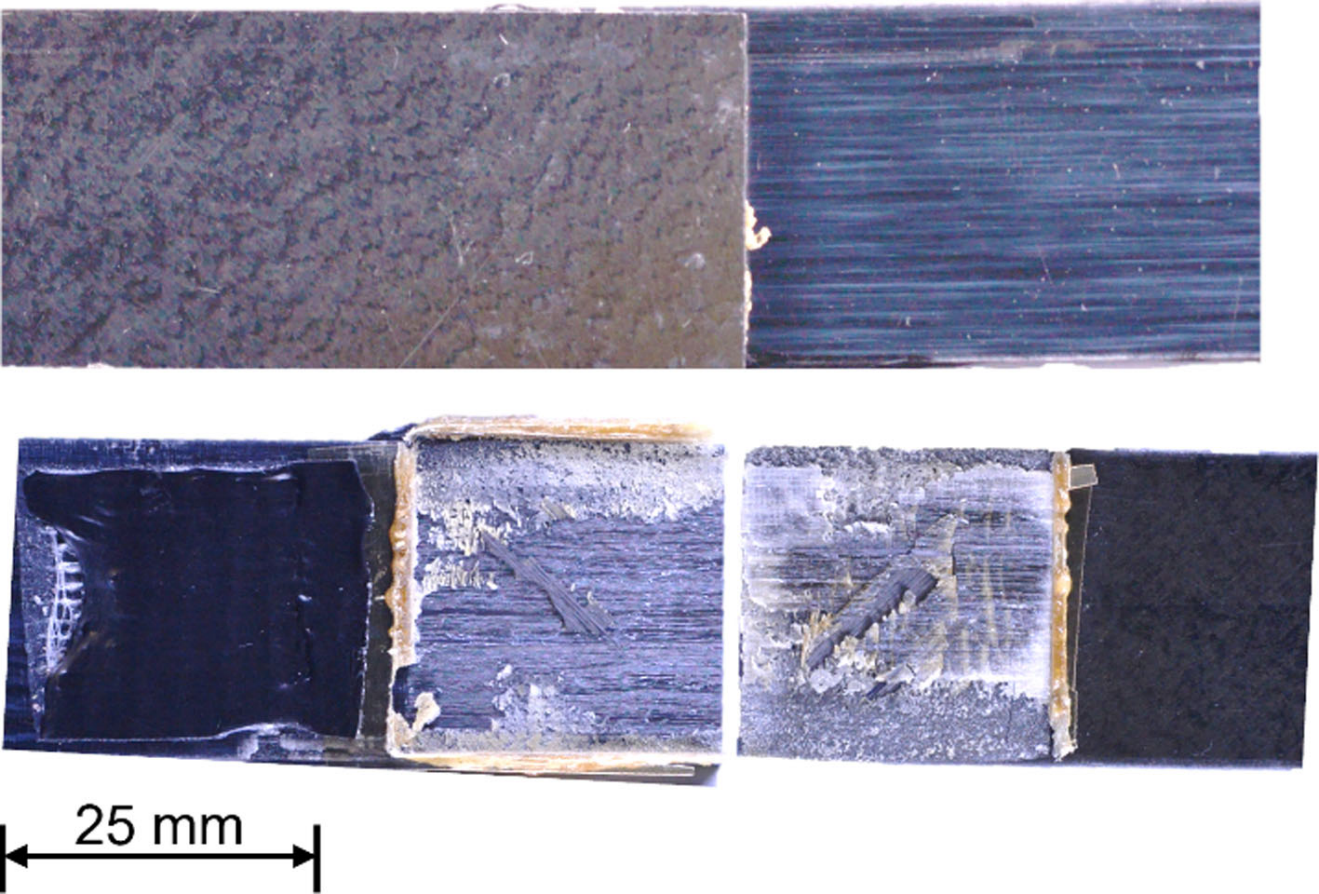
Joint before and after destructive testing produced under the predicted optimal input configuration

To evaluate the model’s ability to optimise the USW process for the dissimilar materials, a complete scan of the bounded parameter space was conducted using the GA–ANN model. Of these predictions, the parameter combinations that yielded the highest LSS while also satisfying class one defects and class one repeatability was determined to be the optimal. The model predicted that the optimal parameters of weld energy = 1.263 kJ, welding force = 842 N and vibrational amplitude = 106 µm will produce a joint with a LSS of 25.3 MPa. The model’s prediction was then subsequently validated by experimentation. Figure 8 displays the joint produced with the optimised process parameters before and after destructive testing. It is evident from the figure that there was complete contact at the joint interface and no un-welded regions, indicating an optimal joint interface with zero defects present (Class 1).

The results of the experiment are in good agreement with the predicted values, where the mean LSS across three experiments using the optimal welding parameters was 24.5 ± 0.3 MPa with RSD = 1.26% (Class 1). A comparison between the predicted and actual values is highlighted in Table 7. The LSS prediction error is just 3%, with 100% accuracy in predicting the correct class for repeatability and process induced defects. Therefore, the robust multi-objective optimisation model developed in this study has optimised the process of joining dissimilar materials through USW. The procedure identified and mitigated the key drawbacks associated with USW, which are process repeatability and process induced defects. It may be noted from Table 3 that only two cases from the DoE (Run 7 and 8) achieved Class 1 defects and repeatability, with the associated LSS of less than 9 MPa, while the maximum LSS achieved in the DoE (25.98 MPa for Run 15) has Class 3 defects.

**Table 7:** Comparison between the predicted and actual responses achieved under optimal conditions

Output	Actual (all)	Actual (average ± SD)	Predicted	Accuracy (%)
LSS (MPa)	[24.11, 24.61, 24.65]	24.5 ± 0.3	25.3	97
Defect class (1–3)	[1, 1, 1]	1	1	100
Repeatability class (1–3)	[1, 1, 1]	1	1	100

### Limitations and future research

The main objective of this study was to demonstrate that a USW process for CF/PEKK to CF/epoxy can be optimised with a 3^3^ DoE dataset in conjunction with machine learning. The results presented in this study indicate that the process has been efficiently optimised using a BO GA–ANN modelling approach. Due to the complex nature of the USW process for the aforementioned dissimilar materials and the limited data available, the authors cannot state with absolute certainty that the BO GA–ANN approach found the true process global optimum. However, the authors can state that relative to the observed DoE data, the optimised process parameters produce a joint of superior performance. Therefore, the BO GA–ANN modelling approach implemented in this study captured the non-linearity in the system and in doing so enabled the process to be optimised to a degree acceptable for the study and application.

The authors recognise the limitations in the pre-planned DoE approach which can produce a sparse dataset. Through ongoing research, the authors are investigating the effectiveness of replacing the pre-planned DoE approach with a discrete Bayesian optimisation (DBO) method, thus creating a sequential optimisation approach. Using the sequential approach, the authors are trialling different machine learning methods for developing surrogate models such as the popular GPR, and neural network Gaussian process models considering input uncertainty, which was developed by Lee et al. (2020) for modelling composite structure assemblies. The effectiveness of the DBO approach will be evaluated against the current study.

## Conclusions

The lap shear strength (LSS) response envelope for the ultrasonic welding scenario implemented in this study is extremely non-linear with respect to process input parameters. It has been found that repeatability and process induced defects are of key concern when optimising the process. Optimising the process using traditional techniques requires multiple DoE’s with refined parameter levels for each DoE iteration. However, the multi-objective optimisation model developed in this study demonstrates the ability to efficiently optimise the LSS using a hybrid GA–ANN model from a single DoE dataset. The multi-objective approach ensures the LSS is optimised while simultaneously accounting for process repeatability and process induced defects. Therefore, this study indicates that hybrid GA–ANN models combined with Bayesian optimisation for hyperparameter tuning, are efficient tools for process optimisation. Although the predictive model developed in this study was focused on USW joints, the model development approach is relevant to the optimisation of any manufacturing process. To our knowledge this is the first application of a hybrid GA–ANN model in conjunction with BO to optimise a dissimilar USW composite joint.
